# Post-stroke dysphagia: identifying the evidence missing

**DOI:** 10.3389/fmed.2025.1494645

**Published:** 2025-02-26

**Authors:** Zicong Wang, Ran Shi, Paulo Moreira

**Affiliations:** ^1^The First Affiliated Hospital of Shandong First Medical University & Shandong Provincial Qianfoshan Hospital, Jinan, China; ^2^Shanghai Sunshine Rehabilitation Center, Tongji University School of Medicine, Shanghai Yangzhi Rehabilitation Hospital, Shanghai, China; ^3^Department of Rehabilitation Medicine, The First Affiliated Hospital of Shandong First Medical University & Shandong Provincial Qianfoshan Hospital, Jinan, China; ^4^Atlantica Instituto Universitario, Gestao em Saude, Oeiras, Portugal; ^5^School of Social Affairs, Henan Normal University, Xinxiang, China; ^6^International Healthcare Management Research and Development Center (IHM-RDC), The First Affiliated Hospital of Shandong First Medical University & Shandong Provincial Qianfoshan Hospital, Jinan, China

**Keywords:** dysphagia, stroke, rehabilitation, healthcare management, geriatrics, elderly

## Abstract

Dysphagia is a high-profile dysfunction that often occurs after a stroke, with a prevalence of 50%−80%. Post-stroke dysphagia (PSD) often leads to serious complications such as pneumonia and malnutrition, reducing the quality of life and leading to poor prognosis or even death. PSD causes these adverse physical and psychological impairments to patients, which becomes a challenge for both patients and physicians. This review intends to contribute to the international debate on evidence-based options on Stroke Rehabilitation and to better understand the need for further research on PSD and summarizing evidence on some of the most relevant topics and clarifying its clinical practice value for Neurology, stroke rehabilitation experts, rehabilitation and nursing staff, as well as patients. The article identifies and discusses the gaps in knowledge on PSD and elaborates on current evidence concerning the selection of subjects, examination methods, patient data extraction and analysis, classification of stroke lesions, details of dysphagia, significance of results, and neuromodulation of dysphagia, from the perspective of rehabilitation physicians. The review identified a set of 10 points and parameters for the international debate on PSD, namely: stroke onset, cognitive impairment, feeding method, contrast medium, swallowing reflex delay, swallowing evaluation form, division of brainstem, multiple stroke sites, basal ganglia lesions and neuromodulation techniques. The article explores available evidence on factors associated with dysphagia and stroke site. Although there is plenty of evidence exploring the correlation between stroke site and swallowing disorders, the pathophysiological mechanisms between the two are complex, and expert interpretations of the evidence and clinical opinions vary on which swallowing abnormalities occur. The study generates evidence on current evidence-based options on Stroke Rehabilitation and a better understanding of the need for further research on Post-Stroke Dysphagia. Taking a patient-centric approach, the ultimate goal is to generate on how can available evidence influence policy or practice or research or clinical education. The article provides a structured discussion clarifying key points on the relationship between stroke lesions and swallowing dysfunctions and contributes to clarifying the gaps in evidence to further improve the quality of life of the patients suffering from Post-Stroke Dysphagia.

## Introduction

Dysphagia is a high-profile dysfunction that often occurs after a stroke, with a prevalence of 50%−80% (1). Post-stroke dysphagia (PSD) can lead to serious complications such as pneumonia and malnutrition, reducing the quality of life and leading to poor prognosis or even death. PSD causes these adverse physical and psychological impairments to patients, which becomes a challenge for both patients and physicians; there is plenty of evidence (2–5) exploring the correlation between stroke site and swallowing disorders. However, the pathophysiological mechanisms between the two are complex, and expert interpretations of the evidence and clinical opinions vary on which swallowing abnormalities occur (6).

Understanding the relationship between stroke site and dysphagia is crucial for developing targeted rehabilitation strategies, improving patient outcomes, and guiding clinical decision-making.

This commentary intends to contribute to the international debate on evidence-based options on Topics in Stroke Rehabilitation and to better understand the need for further research on PSD. We discuss this by summarizing data on some of the most relevant topics and clarifying its clinical practice value for Neurology, stroke rehabilitation experts, rehabilitation and nursing staff, as well as patients. From the perspective of rehabilitation physicians, we will elaborate our views on the selection of subjects, examination methods, data extraction, and analysis, classification of stroke lesions, details of dysphagia and the significance of the result.

We organize our discussion in a set of 10 points briefly introduced in Table 1 and expanded below.

**Table 1:** Factors associated with dysphagia and stroke site.

**Parameter**	
Stroke onset	Within 1 month (5, 7–9)
	Months (2, 10)
Cognitive impairment	Exclusion (5, 7, 37)
	Inclusion (9)
Feeding method	Oral feeding (5, 11, 12)
	Alternative feeding (9, 13–16)
Contrast medium	Semisolid 4 mL bolus (4), 3 mL of water (5)
	2 mL of thin liquid, 5 mL of thin liquid, and 5 mL of curd-type yogurand (12)
	Five types substances (water, puree, banana, soft diet, and cookies) (11)
	5 mL of food puree A and liquid B (3)
Swallowing reflex delay	Delayed if the food was kept inside the mouth for a long period (50)
	Triggered after the bolus reached the pyriform sinuses (5, 54)
	Triggered after the bolus reached the valleculae (55)
Swallowing evaluation form	FOIS (17–19)
	MBSImp (7, 8, 20, 21)
	FEES (75, 76)
Division of brainstem	Brainstem and non-brainstem (22–24)
	Pontine, midbrain, medulla oblongata (8, 25)
	Dorsolateral and medial medulla oblongata (26–28)
Multiple stroke sites	Exclusion (4, 5, 11)
	Inclusion (7, 12, 21, 64)
Basal ganglia lesions	Prolonged pharyngeal bolus transit time (29)
	Severe dysphagia (30, 31)
	Controlled swallowing movements (7)
	prolonged oral transit time (32, 33)
Neuromodulation techniques	5 Hz rTMS (77), 10 Hz rTMS (79)
	5 Hz PES (78)

## Methods

The authors performed a narrative review approach and analysis applying the Scale for the Assessment of Narrative Review Articles–SANRA. The PUBMED, EMBASE and COCHRANE databases were searched for English-language articles, with search terms described as stroke, lesion, dysphagia, swallowing disorders, relationship. Reviews, clinical studies, and case reports were used to contextualize the findings. Articles should meet any of the following criteria:

- Patients with CT/MRI-confirmed stroke;
- Swallowing assessment was completed;
- Stroke sites were categorized;
- The relationship between stroke and swallowing was statistically analyzed;
- Articles involving studies in children (age <18 years) or animals were excluded.

The selection of the period and database is ensured to be relevant to the current clinical practice and research standards. After reviewing the selected topics, two reviewers independently read the abstracts and selected relevant articles for full-text review. Data that met the inclusion criteria were extracted, tabulated, and analyzed. Finally, 81 articles were analyzed. At least two researchers read the selected articles. The researchers arranged the article by topic and created a table for analysis. These articles helped to explain the background of the findings.

Post-stroke dysphagia is a major concern for patients and medical personnel, directly impacting the patient's life and quality of life. Although researchers have conducted studies on this topic, swallowing is a highly delicate and complex motor process, making it challenging to explore the relationship between stroke and swallowing. Therefore, this paper aims to analyze previous articles, refine their findings, exclude studies with inadequate research methods, and suggest more standardized and suitable research methods for future studies.

## Results

The analysis of the included articles exploring factors that may influence the relationship between stroke and dysphagia, as well as the diversity of relationships between the two. Is reflected in the following 10 points:

Point 1. The subjects included in stroke onset studies are usually selected from patients with acute stroke, that is, within 1 month (5, 7–9, 30, 34) after the onset of the stroke. Some acute stroke patients can spontaneously recover the swallowing function in a relatively short period of time; despite this tendency to recover, many stroke patients continue to have swallowing dysfunctions 1 month after stroke (35). Thus, we believe Future studies should take a broader perspective and include patients with acute stroke, and stroke patients who were also in the recovery period (2 weeks to 6 months after the onset). The recovery period is also critical for patients' rehabilitation (36), which will become more relevant for an increasing number of stroke patients. A 2020 retrospective study (2) included patients with a median stroke onset time of 2.5 month s(IQR 0.7–11.0). Significantly, this study indicates that inconsistencies in the length of stroke onset may have had an impact on the results of the study. Thus, additional research is necessary to further analyze the effect of time of onset on swallowing function using multiple regression analysis (5, 34), which seems to be a key idea to be further discussed amongst practitioners worldwide.

Point 2. Cognitive impairment and dysphagia

Most studies on dysphagia exclude patients with cognitive impairment, potentially overlooking the role of cognitive function in post-stroke dysphagia (5, 7, 37). Recent evidence (25, 33) elucidated the clinical features and lesions that contribute to delayed swallowing: lip, tongue and oropharynx correlate with the degree of cognitive impairment, and cognitive function is significantly lower in patients with delayed oral transit time. In addition, lesions in the left frontal lobe were associated with a delayed oral phase. It follows, thus, that consideration of these patients will give additional insights into the factors associated with post-stroke dysphagia and enrich symptomatic diagnosis and treatment.

A study (38) suggested that cognition is better to be considered as a mediator along with physical aspects of dysphagia. Post-stroke dementia or post-stroke cognitive impairment (PSCI) may affect up to one-third of stroke survivors (39). PSCI is closely related to swallowing dysfunctions. The normal ingestion-swallowing process is divided into the anticipatory stage (cognitive stage), oral preparation stage, oral transport stage, pharyngeal stage, and esophageal stage. The anticipatory stage is a prerequisite for oral preparation by recognizing the food's consistency, volume, temperature, and taste to predict the oral handling and routine ingestion procedures. Oral preparation refers to the stage from the intake of food to the completion of mastication, mainly the ingestion and processing of food. These two periods can be controlled at will and stopped at any time. Additional evidence (40) reported that during the anticipatory stage of swallowing, sensory stimuli related to food play an important role in the behavioral and neurophysiological aspects of swallowing. The primary sensorimotor cortex (S1M1) is an important area for executing swallowing as well as integrating sensorimotor information related to swallowing preparation. Cognitive function influences the intake and delivery of bolus during the oral preparation and oral phases. Previous studies have reported that the worse the cognitive function, the higher the prevalence of swallowing dysfunction in stroke patients, and the better the cognitive function, the greater the probability of patients achieving oral feeding (41, 42). Therefore, although available evidence already suggests that PSCI should be prioritized for swallowing assessment and rehabilitation and may be crucial for developing targeted interventions and rehabilitation strategies (43), further evidence is necessary.

Point 3. Inclusion of nasogastric tube-fed patients

Similarly, in a large number of studies on PSD, some patients who have not yet ingested orally are excluded (5, 11, 12); We believe that stroke patients fed by nasogastric tubes should be included, especially to obtain a mechanism for the inability to feed via mouth at different stroke lesions, making the results more valuable for clinical practice. Several studies have included patients requiring tube feeding, considered impaired oral intake and tube feeding as specific signs of dysphagia, and analyzed the site of brain lesions associated with them (9, 13–16). A study in particular (42) identified that 73% of patients who died had alternative alimentation. Therefore, for the patients with PSD who had alternative feeding methods, evidence allows to arugue that clinical intervention should actively implement intervention measures as soon as possible. Those who have not yet eaten orally are not unable to do so but are unable to do so safely. Hence the need for additional evidence on when to add to clinical procedures followed by Rehabilitation professionals on when to perform VFSS on these patients to assess their safety in eating and formulate a rehabilitation plan for when and how to begin oral intake assuming the best swallowing treatment is swallowing.

Point 4. Consistency and volume of swallowing contrast solvents

In this point we want to contribute to the international debate by pointing out that there is evidence to sustain the argument that consistency and volume of the swallowing contrast solvent are not the same; hence, the possibility that this may contribute to the different findings between stroke site and VFSS findings, which may be more one-sided for some studies using only one solvent (4, 5). For example, a particular study (12) used the three substances: 2 mL of thin liquid, 5 mL of thin liquid, and 5 mL of curd-type yogurand to patients swallow, suggesting that increasing the bolus viscosity may reduce the aspiration severity. Another study (11) used five types substances (water, puree, banana, soft diet, and cookies) to test the relationship between stroke lesions and aspiration. On other study (3) took 5 mL of food puree A and liquid B as the solvents for VFSS, and no correlation was observed between the pharyngeal response time (PRT) and lateralization of brain lesion. We believe that Future studies should include liquids with different volumes, such as 7 mL, 10 mL, or with different viscosity of thin liquids or thickened liquids, thus providing details of what properties cause swallowing disorders at different stroke areas, or what kind of swallowing disorders occur at the same stroke area with different properties, thus making new available evidence for practice more comprehensive.

Hence, the decision of using iodine contrast medium liquids is usually determined based on the results of clinical evaluation (44–47). In principle, the amount of liquids is from less to more, from thick to thin, as thin liquids or water may delay the swallowing reflex in patients. Sensory feedback plays an important role in the oral stage, for patients with bucco-facial apraxia after stroke, the sensory pathway of the oral proprioceptor and tactile mechanoreceptor into the central nervous system (CNS) is damaged and the signal transmission is delayed, while effective coordination of these jaw and tongue movements depends on the integration of information from a dense array of sensory receptors in the oral (48), thus leading swallowing uncoordinated and prone to aspiration as water is easily deformable and flowable. Some studies advocated that thickened liquids have a shorter laryngeal vestibule closure time than thin liquids and that thickened liquids do not increase pharyngeal residue and are safer for patients (46, 49–51). Thus, to what extent examining the different properties of the liquids may refine the association between stroke lesions and swallowing dysfunctions details and may provide guidance for the development of a swallowing dysfunction diet? This is another topic we humbly suggest for further international debate and additional generation of evidence. When it comes to the accuracy of dysphagia assessment, it is important to mention flexible endoscopic evaluation of swallowing (FEES), it is only considered the other gold standard (next to VFSS). The sensitivity of the FEES to aspiration and pharyngeal residue was higher than that of the VFSS as derived from the Giraldo (75) study. The sensitivity of the two tests to detect premature pharyngeal spillage was similar. However, it should be taken into account that the FEES may be more uncomfortable for the patient to use, as well as the 'white screen' at the moment of swallowing, not being able to see the video during swallowing is also a disadvantage of the FEES. The Espitalier (76) study demonstrated that the VFSS allows better quantification of pharyngeal residues. The VFSS is relatively more commonly used in the clinic, and objective evaluation of the observation form becomes crucial.

Point 5. Interpretation of VFSS and swallowing reflex delay

The interpretation of VFSS varies significantly, the more controversial point being the determination of the swallowing reflex delay. Some studies do not have a clear definition of swallowing reflex delay. For example, one study (52) considers the food kept inside the mouth for a long period as a delay, whereas in other study (53) patients with a latency time >3 s were defined as having a delayed swallowing reflex. Additional International evidence reports different definitions of delayed swallowing reflex. Two relevant studies (5, 54) defined it as liquid remaining in the pyriform sinuses for more than 0.1 s (3 frames) before swallowing. Another study (55) generates evidence to argue that the delayed swallowing reflex is triggered 1 s after the bolus reached the valleculae. However, the hyoid elevation is often used as a marker for the initiation of the swallowing reflex under VFSS. Hence, two other studies (56, 57) define the starting point of pharyngeal swallowing as the head of the bolus reaching the lower edge of the mandibular branch; the end point is the last video frame from the head of the bolus to the vallecular sinus, until the hyoid bone is raised. Thus, further research is required to clarify how the swallowing reflex delay may need to be unified through the frame by frame analysis of VFSS to reduce the risk of misjudgment.

Point 6. Patterns of PSD abnormalities

There are a variety of patterns of PSD abnormalities, with most studies including aspiration and laryngeal penetration, clearance or prevalence of oral cavity residue, vallecular residue, pharyngeal residue after one swallow, and swallowing reflex delay (2, 4, 11, 12). However, generation of evidence could be more comprehensive. Several studies (7, 8, 20, 21) applied the Modified Barium Swallow Study Impairment Profile (MBSImP) to analyze the relationship between brain lesion location and 17 physiological aspects of swallowing. The results of these studies suggest that laryngeal elevation, anterior hyoid excursion, laryngeal vestibular closure, and pharyngeal residue can be associated with lesioned voxels or regions of interests. It seems like when the VFSS videos were analyzed using the MBSImP, the results were more accurate and could include more details of swallowing dysfunctions. As a standardized scale, MBSImP (58) information gained from the examination is critical for identifying and distinguishing the type and severity of swallowing impairment, determining the safety of oral intake, testing the effect of evidence-based frontline interventions, and formulating oral intake recommendations and treatment planning. The swallowing movement is one of the most complex and unique movements of the body, involving sequential activation and deactivation of the oropharyngeal muscles. It involves the contraction of the submental muscle groups, the upward and forward movement of the hyoid bone, the epiglottis folding back, the contraction of the pharyngeal constrictor muscles, the opening of the cricopharyngeal muscle and other important steps to ensure the closure of the airway and the opening of the esophagus.

MBSImP includes 17 important physiological elements of the swallowing process. We purpose therefore, for the international debate on the matter, that Future studies should undertake scoring details within the MBSImp (59–61), such as lip closure, soft palate elevation, and laryngeal elevation can all be used to enrich the system for the relationship between stroke lesions and swallowing dysfunction patterns. Laryngeal elevation plays two important roles: one is to achieve airway protection; the other is to pull the cricopharyngeal muscle forward and promote its opening. Once the laryngeal elevation is abnormal in time or degree, it will cause bolus to enter the airway through the throat, resulting in aspiration. Therefore, we can argue that each step of swallowing deserves special specific attention in Future research.

Point 7. Brainstem stroke and dysphagia

There are several studies on the relationship between brainstem stroke and dysphagia. However, the division of brainstem sites varies, with some divided into brainstem and non-brainstem (22–24), others into pontine, midbrain, medulla oblongata (8, 25), and others into dorsolateral and medial medulla oblongata (26–28). In this context, we argue that we need more international evidence to allow for a better understanding of the implications when we divide brainstem into the midbrain, pons, medial medulla, and lateral medulla. The brainstem, as a vital center containing various important reflex centers, is connected to the III-XII cranial nerves and is a relay station for the superior and inferior afferent pathways. The brainstem swallowing center is located in the medulla oblongata, called the medullary central pattern generator (CPG) (62), and when it is damaged, the nucleus tract solitaries-dorsal swallowing group (NTS-DSG) is unable to synthesize the afferent information and affects the initiation of swallowing patterns; the medulla-ventral swallowing group (VLM-VSG) fails to distribute swallowing drive to the various motor nerve pools associated with swallowing, affecting the motor drive of cranial nerves V, VII, IX, X, and XII; excessive salivation; difficulty in laryngeal elevation; retention of food in the vallecula sinus; aspiration; or even in severe cases, not knowing how to swallow; and cricopharyngeal achalasia. On study (10) concluded that the medullary region governs the rhythmic pattern of pharyngeal swallowing; whereas the pontine region transmits the received peripheral information upwards to the CNS. Furthermore, another study (63) uncovered the importance of the primary motor cortex-parabrachial nuclei and nucleus tractus solitary (M1-PBN-NTS) neural circuit in driving the protective effect of electroacupuncture (EA) stimulation at the CV23 acupoint (EA-CV23) against swallowing dysfunction and thus reveal a potential strategy for dysphagia intervention. Therefore, evidence on best approaches to achieve more precise analysis of swallowing dysfunction seems necessary. Hence, new appropriate therapeutic approaches could be subsequently applied to the stroke lesions.

Point 8. Multi-site stroke and swallowing outcomes

Some studies (4, 5, 11) have excluded multiple stroke site (2 or more). While other studies (7, 12, 64) incorporate multi-site stroke cases, summarizing that lesions located in supratentorial and infratentorial regions (i.e., multiple sites) were predictive of poor swallowing outcomes, mainly including primarily the sensorimotor integration areas and their corresponding white matter tracts. We believe that stroke patients with multiple lesions should be included in Future studies, as multiple lesions may exacerbate swallowing disorders. One study (21) suggested that the combination of lesioned regions might also determine the recovery of swallowing function. For example, combined strokes in insular and frontal regions are independent predictors of prolonged dysphagia course. More evidence on this point is required. Including such patients in Future studies may provide more mechanisms for CNS control of swallowing, as well as diverse clinical manifestations of swallowing disorders in patients, for timely detection and treatment.

Point 9. Basal ganglia lesions and silent aspiration

Different views on the characteristics of PSD caused by basal ganglia lesions have been studied (7, 9, 29–33). Basal ganglia lesions are significant independent factors for swallowing reflex delay. One other study (65) has reported that basal ganglia infarction leads to impaired dopamine metabolism and decreased production of substance P (SP), decreased SP concentration in the pharynx and tracheal mucosa, and decreased pharyngeal and cough reflexes, making it very easy for aspiration to occur. More importantly, it is a silent aspiration (especially when it occurs during sleep, without coughing), which leads to aspiration pneumonia. The swallowing and coughing reflexes are the defense mechanisms that prevent the inhalation of pharyngeal contents into the lower respiratory tract. It has also been reported (66) that the basal ganglia infarction is associated with attention deficit and bucco-facial apraxia. Therefore, disuse is also one of the important features of basal ganglia lesions, and damage to the basal ganglia area not only has a delayed pharyngeal transmission time but may also trigger dangerous silence aspiration. Silence aspiration is extremely harmful to patients and deserves our attention for further investigation in Future studies as also proposed in recent studies (67–70) and following recent trends in healthcare research (71–74).

Point 10. Neuromodulation techniques for dysphagia

Neuromodulation techniques for dysphagia are commonly used, such as pharyngeal electrical stimulation (PES), repetitive transcranial magnetic stimulation (rTMS), etc. For the site and intensity of stimulation is the focus of experts' research. Twelve patients with dysphagia were randomly divided into 5 Hz rTMS, 1 Hz rTMS, or PES for neuromodulatory technology intervention (77). The patients were assessed by VFSS before and 60 min after the intervention to calculate the penetration aspiration scores (PAS). In the 5 Hz rTMS and PES intervention groups were shown to stimulate cortical excitation within the swallowing motor system, 1 Hz transcranial magnetic stimulation resulted in cortical inhibition, and the 5 Hz frequency stimulates the motor function of the pharyngeal mucosa, which led to a better induction of excitability in the cerebral swallowing motor cortex. Due to the small number of patients, it was not possible to compare the effectiveness between the different interventions. And PAS, which is not a perfect assessment of swallowing function. Future larger studies are needed to further explore the efficacy of these neuromodulatory treatments for dysphagia. Suntrup-krueger (78) found that PES could improve swallowing function by promoting increased secretion of neuropeptide substance P in saliva and enhancing the swallowing reflex by studying 20 healthy volunteers who underwent PES for 10 min 5 Hz. However, the experiment did not perform VFSS in patients with dysphagia as a means of demonstrating that PES improves swallowing function. Du's study concluded that 10 Hz rTMS is effective in the treatment of PSD, and that the C3 (left the central) area [86] may be a target for rTMS in the treatment of PSD. However, the assessment of swallowing function was missing the esophageal phase, as well as there was no detailed explanation for the C3 region. Therefore, larger and precise studies on the neuromodulation of dysphagia are needed in the future to explore innovative therapeutic targets to improve efficacy.

## Conclusions

This literature review contributes to the international debate on evidence-based practice for recognizing the relationship between stroke and dysphagia. The review identified ten key points and parameters related to PSD, including stroke onset, cognitive impairment, feeding method, contrast medium, swallowing reflex delay, swallowing evaluation form, division of brainstem, multiple stroke sites, basal ganglia lesions, and neuromodulation techniques. The main findings emphasize the need to standardize the assessment of dysphagia using VFSS, improve the classification of stroke lesions, and refine the relationship between stroke and dysphagia to guide treatment.

Regarding evidence-based practice in identifying the relationship between stroke and dysphagia. Summarized with the following:

- Standardize the process of assessing dysphagia with the VFSS;
- Make the classification of stroke lesions more complete;
- Refine the relationship between stroke and dysphagia to make the results more informative and thus guide treatment.

For clinical practice, it provides a comprehensive framework for evaluating and managing post-stroke dysphagia. By identifying key points and parameters related to PSD, it equips clinicians with a more nuanced understanding of the condition, enabling them to tailor treatment plans to individual patient needs. Furthermore, the emphasis on standardizing the assessment process with VFSS and refining the relationship between stroke and dysphagia can lead to more effective interventions, reducing complications and improving patient outcomes. Ultimately, this study contributes to enhancing the overall quality of stroke care and rehabilitation, fostering a more patient-centered and evidence-based approach to managing post-stroke dysphagia.

This article also has some limitations, firstly, the article only analyzed patients whose primary disease was stroke, and did not analyze swallowing dysfunction caused by other diseases, a wider range of diseases can be included in the next step; secondly, the article only discussed the specific matters of the VFSS assessment of swallowing dysfunctions, with the development of swallowing assessment tools, the laryngoscopy, high-density surface EMG, and other instrumental assessments should be studied as well; lastly, our article only analyzed the treatments of swallowing dysfunctions of Neuromodulation techniques, and in the future, more treatments can be included in the analysis. Another limitation was that limited results are described for neuromodulation techniques, although numerous studies have been carried out in recent years, and this is because the focus of the study was set on other techniques; the number of patients described in the present review is very limited and transcranial direct current stimulation (tDCS) was not considered.

Future studies may tackle the Limitations points identified and based on clinical and imaging factors, including the selection of the study population, the distribution of stroke types, and the assessment of the details of dysphagia, and we emphasize the importance of these factors as prognostic factors. Clinicians and rehabilitation professionals should further consider the broad spectrum of mechanisms of recovery and prognosis of PSD to support the development of neuroanatomical models of PSD physiology and therapeutic approaches that address the neurophysiologic basis of PSD as well as neuromodulation techniques.
